# L-carnosine as an enhancer to computerized cognitive behaviour therapy in Japanese workers, an unjustified claim

**DOI:** 10.1186/s13030-018-0130-3

**Published:** 2018-09-04

**Authors:** Geneviève Sauvé, Marie-France Bastien, Casandra Roy-Gelencser, Ghassan El-Baalbaki

**Affiliations:** Département de psychologie, Université du Québec à Montréal, C.P. 8888 succursale Centre-ville, Montréal, Québec H3C 3P8 Canada

**Keywords:** Psychological well-being, Fatigue, Anxiety, Supplement, L-carnosine, Computer-based cognitive-behavioural therapy (CCBT), Clinical significance

## Abstract

This letter comments on the conclusion drawn by Shirotsuki et al. (2017) in their article entitled “The effect for Japanese workers of a self-help computerized cognitive behaviour therapy program with a supplement soft drink”, recently published in *BioPsychoSocial Medicine*. The authors concluded that their drink, containing L-carnosine, enhances the effects of a computerized cognitive-behavioural therapy (CCBT) on the psychological well-being of healthy Japanese workers. Yet, we argue that their conclusion is unfounded given their results and the methodological shortcomings of their study. Briefly, while the authors reported improvement on the tension-anxiety subscale of the *Profile of Mood States* (POMS) in the CCBT only group, they also observed a lack of improvement on this subscale in the CCBT+L-carnosine group suggesting that the drink washes out this beneficial effect of CCBT. Methodological issues include the uncontrolled levels of L-carnosine metabolized by participants jeopardize the study’s internal validity. Also, the clinical meaningfulness of the findings seems dubious as post-treatment scores remained within the range of the general Japanese population. Consequently, we argue that Shirotsuki et al.’s study should be re-conducted before drawing any valid conclusion.

Shirotsuki et al.’s study [1] assessed whether the intake of a carbonated drink containing L-carnosine increases the effects of a computerized cognitive-behavioural therapy (CCBT) on psychological well-being of healthy Japanese workers. To achieve their goal, the authors conducted a randomized-controlled trial with three groups: an experimental group (*n* = 29) receiving the dietary supplement in combination with the CCBT, a first control group (n = 29) receiving only CCBT, and a second control group (n = 29) receiving neither of the two forms of intervention. The CCBT used in this study is a six-week psychoeducational personal development program addressing stress management and coping via behavioral activation and cognitive restructuring. The dietary supplement used in this study, the L-carnosine molecule, is a widely studied dipeptide with antioxidant properties. Participants of the study consumed 200 mg of L-carnosine in a 100 ml soft drink each morning during the 6 weeks of intervention. Psychological well-being was operationalized via measures of anxiety, depression and fatigue levels. Additional measures of self-efficacy as well as medical and psychosomatic symptoms were also reported. Following their analyses, the authors concluded that consuming a soft drink containing 200 mg of L-carnosine increased the effectiveness of the CCBT on fatigue levels. However, we believe that the authors’ conclusion is unfounded given their results and the methodological shortcomings of the study that we will detail below.

Beforehand, we wish to acknowledge the authors’ transparency regarding the involvement of Suntory Global Innovation Center Limited in the study’s funding and execution, as well as the multiple roles held by two of their employees as authors (YN and KA) and creators of the drink containing L-carnosine. In the study, it was also reported that participants were employees of a Suntory Group’s production factory. We thought important to remind the reader about the context in which this experimental research took place, given the high threat to internal validity caused by the well documented investigators’ bias as well as the subjects’ and participation biases [2–4], making the quality of this study low as per the *Cochrane Risk of Bias Tool for Randomized Controlled Trials* ([5], Table 8.5.d). More precisely, in Shirotsuki et al.’s study [1], we found a high risk of bias in selective reporting, incomplete outcome data, and blinding of participants and outcome assessment, meaning that conclusions of such low quality studies must be cautiously taken.

Our first methodological question, refers to the uncontrolled consumption of L-carnosine by participants in their regular diet. The verification of L-carnosine can be made using High Performance Liquid Chromatography (HPLC) on urine or plasma samples [6]. The lack of doing so causes a threat to the internal validity: the improvement observed in the L-carnosine group on the POMS-fatigue subscale could be explained by the participants’ regular diet and not the drink. As indicated by the authors, significant amounts of L-carnosine are present in meat (e.g., poultry and beef), which can promote recovery following a state of mental fatigue. Alternatively, 1- participants’ diet can be assessed longitudinally using a food-intake journal, 2- they can be asked to maintain their dietary habits throughout the study, and/or 3- background diet (e.g., vegetarianism, dietary supplements intake) can be used as an exclusion criterion as suggested by Woodside, et al. [7].

Another question we have for the authors, has to do with the clinical significance associated with the reported statistically significant results. How come the authors did not calculate a reliable change index, classically proposed by Truax and Jacobson in 1991 and Speer in 1992 [8], in order to grant that these statistically significant changes reflect a clinically meaningful improvement and not only a statistical regression towards the mean. A number of external factors could therefore explain the observed group differences. Moreover, the post-treatment means on the ‘tension-anxiety’ and the ‘fatigue’ POMS subscales, remain within the range of scores exhibited by the general Japanese population (mean ± 1SD) [9]. Hence, the statistical improvement reported, could be the result of participant’s bias (acquiescence response style) [2]. Further, the psychometric validation study of the Japanese version of the POMS used a sample constituted only of men and did not report any clinical threshold or cutoffs [10]. Consequently, without a clear indication that the gains observed following the intervention had a noticeable clinical impact in participants’ lives [2], it becomes problematic to claim that CCBT improves the subjective perception of well-being and that L-carnosine increases such effects. Which, again calls for the calculation of a clinically reliable index. It is safe to conclude that implementing such a program is yet unwarranted, given that the post-treatment scores do not distinguish the trial participants from what is observed in the general Japanese population [9].

Finally our last remark, is about the lack of improvement on the tension-anxiety subscale in the combined group (CCBT+L-carnosine), whereas the CCBT group without drink, had shown improvement (Cohen’s d effect size = .37) ([1], p.5). At first glance - if we dismiss all the methodological issues raised that could render this result non valid - we could conclude that adding a L-carnosine drink to CCBT is deleterious to CCBT for stress management! In other words, the addition of the authors’ mother industry drink to CCBT, reduces fatigue but washes-out the beneficial effect of CCBT on tension-anxiety. Tension and anxiety are correlated to stress, and tension, anxiety and stress are correlated to fatigue. So here are some additional questions: 1- authors reported that a measure of treatment adherence was completed but no results were given. How did the participants of the CCBT+drink compare to the CCBT only protocol? 2- if tension-anxiety is not enhanced, how long the fatigue improvement will last, given that prolonged presence of tension and anxiety leads ultimately to fatigue. 3- What is the rationale of a stress management treatment that does not address all main symptoms and causes?

In conclusion, we wish to acknowledge that Shirotsuki, et al. [1] reported the limitations of their study (e.g., absence of a control group with a placebo drink, absence of a control group with the L-carnosine drink only, social desirability of participants). Nevertheless, these limits remain highly threatening to the internal validity and our questions relative to what looks like serious methodological shortcomings should lead to only one conclusion: the study of Shirotsuki, et al. [1] should be re-conducted before any valid conclusion could be drawn.

## Abbreviations

CCBT: Computerized cognitive-behavioral therapy
POMS: Profile of Mood States
HPLC: High Performance Liquid Chromatography
POMS-TA: POMS tension/anxiety subscale
POMS-F: POMS fatigue subscale

## References

[1] Shirotsuki K, Nonaka Y, Abe K, Adachi SI, Adachi S, Kuboki T, Nakao M (2017). The effect for Japanese workers of a self-help computerized cognitive behaviour therapy program with a supplement soft drink. Biopsychosoc Med.

[2] Kazdin AE (2016). Research design in clinical psychology.

[3] Paulhus DL (1984). Two-component models of socially desirable responding. J Pers Soc Psychol.

[4] Keeble C, Barber S, Law GR, Baxter PD (2013). Participation bias assessment in three high-impact journals. SAGE Open.

[5] Shuster JJ. Review: Cochrane handbook for systematic reviews for interventions, version 5.1.0, published 3/2011. In: Higgins JPT, Green S, editors. The Cochrane Collaboration, 2011. Available at: www.cochrane-hand-book.org.

[6] Park YJ, Volpe SL, Decker EA (2005). Quantitation of carnosine in humans plasma after dietary consumption of beef. J Agric Food Chem.

[7] Woodside JV, Welch RW, Patterson CC, McKinley MC, Lovegrove JA, Hodson L, Sharma S, L-NS A (2015). Study design: intervention studies. Nutrition research methodologies.

[8] Speer DC (1992). Clinically significant change: Jacobson and Truax (1991) revisited. J Consult Clin Psychol.

[9] Yokoyama K, Araki S, Okajima F, Nomura S, Okuyama I. Examination of the Japanese edition of profile of mood states (POMS) and short versions. Jpn J Public Health. 1993;40:1055. [in Japanese]

[10] Yokoyama K, Araki S, Kawakami N, Kakeshita T (1990). Production of the Japanese edition of profile of mood states (POMS): assessment of reliability and validity. Nippon Koshu Elsei Zasshi.

[11] Tanaka M, Kamohara R, Fujii H, Hirayama Y (2008). Effect of CBEX-Dr-containing Drink on Physical Fatigue in Healthy Volunteers. Jpn Pharmacol Ther.

[12] Shimizu K, Fukuda M, Yamamoto H (2009). Effect of repeated intake of imidazole dipeptides-containing drink on healthy people with feeling of fatigue from daily activities —the results of 207 volunteers enrolled in the 1^st^ recruitment—. Jpn Pharmacol Ther.

[13] Yamano E, Tanaka M, Ishii A, Tsuruoka N, Abe K, Watanabe Y (2013). Effects of chicken essence on recovery from mental fatigue in healthy males. Med Sci Monit.

[14] Yokoyama K, Shimomitsu T, Nomura S (2002). Shindan Shido ni ikasu POMS jirei syu (in Japanese).

[15] Oka T, Tanahashi T, Chijiwa T, Lkhagvasuren B, Sudo N (2014). Isometric yoga improves the fatigue and pain of patients with chronic fatigue syndrome who are resistant to conventional therapy: a randomized, controlled trial. Biopsychosoc Med.

[16] Yoshihara K, Hiramoto T, Sudo N, Kubo C (2011). Profile of mood states and stress-related biochemical indices in long-term yoga practitioners. Biopsychosoc Med.

[17] Fukuo W, Yoshiuchi K, Takimoto Y, Sakamoto N, Kikuchi H, Hachizuka M (2008). Comparison of temporal changes in psychological distress after hematopoietic stem cell transplantation among the underlying diseases of Japanese adult patients. Biopsychosoc Med.

