# Supplementary material for: L-carnosine as an enhancer to computerized cognitive behaviour therapy in Japanese workers, an unjustified claim
Source: Biopsychosoc Med. 2018 Sep 4;12:11. doi: 10.1186/s13030-018-0130-3 (PMC6122662; doi:10.1186/s13030-018-0130-3)
Supplement: Related texts

# Reply to the comment on L-carnosine as an enhancer of computerized cognitive behaviour therapy in Japanese workers

**Authors:** Kentaro Shirotsuki, Yuji Nonaka, Keiichi Abe, So-ichiro Adachi, Shohei Adachi, Tomifusa Kuboki, Mutsuhiro Nakao

**Affiliations:** 2 0000 0001 0356 8417 grid.411867.d Faculty of Human Sciences, Musashino University, Tokyo, Japan; 3 Innovation Development Department, Suntory Global Innovation Center Limited, Osaka, Japan; 4 grid.482562.f National Institute of Health and Nutrition, Tokyo, Japan; 5 Medical corporation So-bun-kai, Clinic Adachi, Gifu, Japan; 6 0000 0001 2151 536X grid.26999.3d Professor Emeritus, Tokyo University, Tokyo, Japan; 7 0000 0004 0531 3030 grid.411731.1 Faculty of Medicine, International University of Health and Welfare, Tokyo, Japan

## Abstract

This article responses to the comments on the study entitled “The effect for Japanese workers of a self-help computerized cognitive behavior therapy program with a supplement soft drink” [1]. Certain limitations of the study, such as participants, measurements, and the study protocols among others have been pointed out in these comments. However, the possible limitations of the research were explicitly addressed in the original article [1] and future tasks were discussed before making any conclusions. It was suggested that future studies should use a larger sample of participants from independent institutions and communities to confirm our results. We also strictly evaluated compliance by conducting the program as objectively as possible after randomization. However, more efforts are needed to implement more rigorous designs in the future. Moreover, because many factors are known to differentially affect each mood state, we individually discussed mechanisms that decrease anxiety and fatigue. The strength of the study is that all the procedures were described to facilitate re-testing by other investigators and the results of all the measured variables were shown, regardless of whether they were statistically significant or not. It is suggested that more investigators should be involved in examining additional effects of supplements on cognitive behavioral intervention.

**Keywords**: Computerized cognitive behaviour therapy, Workplace, L-carnosine, Self-help

In the original study [1] we examined whether a supplement drink that includes L-carnosine enhances the effect of CCBT on psychological well-being. The results suggested that the self-help in the CCBT program reduced the subjective experience of tension-anxiety in a group of workers. The addition of a supplement drink enhanced the effect of CCBT on fatigue. Therefore the CCBT program and the addition of a supplement drink was considered one possible approach to enhancing the effects of CCBT programs on anxiety and fatigue. An article commenting on the study has pointed out certain problems and limitations of this research. However before marking the final conclusions of the original study, we described the same limitations and explicitly addressed them

## Participants

The Suntory Global Innovation Center Limited supported this study and two of their employees were the authors (YN and KA) and the developers of the drink containing L-carnosine. We also reported that study-participants were employed in a Suntory Group factory. We pointed out in the section on the limitations of the study in the original article that the support given by the Suntory group for this study and having Suntory employees as participants was a limitation of the study and that there was room for improving the study. Nevertheless, employees of the division responsible for this study did not participate in the study. Additionally, a simple randomization procedure was conducted by an independent study controller who was not an employee of the Suntory Global Innovation Center Limited and had no direct contact with the participants. Therefore, the issue concerning the selection of participants, we took all possible measures to ensure the reliability of the study.

Moreover, there was no arbitrariness in reporting the study results because we reported all the results including those that were not statistically significant. Furthermore, an independent third party was in charge of data analysis and missing values among others were properly processed. Also, all the indications suggest that the data were complete. Furthermore, because this research involved a company, we clearly stated all the necessary information including the COI disclosure. However, blind participation procedures are impossible in CBT studies. As a result, we did not consider using blinding and randomizing procedure when drinking the beverage because the main objective of this study was to test the effects of CBT. Therefore, it is suggested that a large-scale study be undertaken in the future with participants from outside the Suntory corporation and also include a procedure for blindly drinking the beverage.

## Consumption of L-carnosine and daily dietary records

As indicated in the comments, it was important to correctly check the participants’ regular diet and eating habits. Therefore, in the study, we also asked the participants to regulate their diet and eating habits at the time of requesting their consent to maintain lifestyle habits and refrain from overeating and overdrinking during the program. Moreover, we analyzed the data of participants with a supplement drink intake rate higher than 90%. These procedures were similar to previous works [11–13]. In this study, we also conducted randomization to ensure that dietary habits of the three groups were identical. In addition to teaching the development of lifestyle habits as a part of the CCBT program, we also recorded the meals of the control group and conducted minimal management. The study by Shirotsuki et al. [1] was done mainly to conduct and assess the CCBT program. In the study, the participants recorded data on their daily life activities, and the programs that they followed on worksheets. We did not ask the participants to strictly control and record their diet because it might have placed a heavy burden on the participants and resulted in dropouts and noncompliance. Moreover, the main assessments of the study were psychological indices and no physiological indices were used. It is suggested that future studies should assess physiological indices to clarify overall changes.

The measurement of physiological changes is a necessary task for the future. However, in the study by Shirotsuki et al. [1] the participants experienced a minimal degree of control. Also, excessive recording and management might have decreased the motivation and increased the drop-out rate from the CCBT program. Therefore, in this study, we did not require that participants perform too many tasks or recordings to ensure their compliance.

## POMS

It is true that the cited article on POMS [14] is based only on male participants. However, the Japanese version of POMS has been analyzed with an enormous amount of data of healthy and clinical samples of men and women, and the scores have been standardized. POMS is widely recognized as useful in Japan, and it has well demonstrated clinical significance [14]. Several BioPsychosocial Medicine studies [15–17] have also used POMS. In recent years, POMS-2 has been developed and refined. POMS has been properly standardized, but additional refinements including measurements on different scales could be useful.

The study by Shirotsuki et al. [1] was a pilot study conducted with healthy participants to assess psychological traits of the participants. In the study, participants with mental or physical health risks and those with high POMS scores were excluded. It would be necessary to investigate participants with psychological or mental health problems to establish the clinical significance of the study. Nevertheless, Shirotsuki et al. suggested primary preventive measures based on the investigation of healthy participants.

## Compliance and anxiety and fatigue

To assess the compliance with the program, participants with a drink intake rate of less than 90%, those that maintained a life record less than 90% of the time, those that conducted the CBT task less than 90% of the time, those that did not complete watching the CBT videos six times, and those showing considerably large variations in their life records of sleep among others were excluded from the analysis. The subjects of analysis were participants with a high performance who satisfied these conditions. Therefore, the compliance of the subjects in this study was high.

Although fatigue and anxiety might be related, there are many factors that affect fatigue. Fatigue and anxiety might not change in the same way. There is little clear evidence that anxiety and fatigue in this study were closely related. From supplementary data of the correlation between TA and F in the CCBT + Drink group, it is difficult to conclude that *r* = .28 indicate a strong relationship with each other. Supplementary reporting, in the CCBT + Drink group, the effect size of POMS-TA was as low as d = .19. The comments suggested the possibility that the supplement drink might inhibit the reduction of anxiety. However, based on the data above, we do not agree with this suggestion. Moreover, the CCBT program conducted in this study was a Self-Help program. Participants themselves could have dealt with the part of the program related to stress management. Moreover, the program was rather simple, and it was possible to complete it in only six sessions. Therefore, its effect might not have been significant. The results might be improved by increasing the number of sessions and support provided by therapists, which would make the findings of this study more robust. Generally, CCBT programs are more effective for anxiety and depression, rather than for physical symptoms, which might have affected the results of this study.

## Conclusion

There are limitations to this study, and future studies are needed to clarify specific issues. Nevertheless, this study suggested the possible positive effects of simultaneously using a CCBT program and L-carnosine. Future studies could improve the design of this study by including a placebo drink group. Moreover, using a different research design could improve the CCBT program. Furthermore, different researchers should conduct this investigation in the future with different participants. Shirotsuki et al. [1] used randomization procedures; however, there were between-group differences in specific indices. Therefore, it might be possible to obtain clearer results by developing research designs that eliminate between-group disparities.
